# Pragmatics in the False-Belief Task: Let the Robot Ask the Question!

**DOI:** 10.3389/fpsyg.2020.593807

**Published:** 2020-11-23

**Authors:** Jean Baratgin, Marion Dubois-Sage, Baptiste Jacquet, Jean-Louis Stilgenbauer, Frank Jamet

**Affiliations:** ^1^Laboratoire Cognition Humaine et Artificielle, Université Paris 8, Paris, France; ^2^Probability, Assessment, Reasoning and Inferences Studies (P-A-R-I-S) Association, Paris, France; ^3^Facultés Libres de Philosophie et de Psychologie (IPC), Paris, France; ^4^CY Cergy-Paris Université, ESPE de Versailles, Paris, France

**Keywords:** theory of mind, preschool children, pragmatics, humanoid robot, mentor-child context, ignorant robot, human robot interaction, first-order false belief task

## Abstract

The poor performances of typically developing children younger than 4 in the first-order false-belief task “Maxi and the chocolate” is analyzed from the perspective of conversational pragmatics. An ambiguous question asked by an adult experimenter (perceived as a teacher) can receive different interpretations based on a search for relevance, by which children according to their age attribute different intentions to the questioner, within the limits of their own meta-cognitive knowledge. The adult experimenter tells the child the following story of object-transfer: “Maxi puts his chocolate into the green cupboard before going out to play. In his absence, his mother moves the chocolate from the green cupboard to the blue one.” The child must then predict where Maxi will pick up the chocolate when he returns. To the child, the question from an adult (a knowledgeable person) may seem surprising and can be understood as a question of his own knowledge of the world, rather than on Maxi's mental representations. In our study, without any modification of the initial task, we disambiguate the context of the question by (1) replacing the adult experimenter with a humanoid robot presented as “ignorant” and “slow” but trying to learn and (2) placing the child in the role of a “mentor” (the knowledgeable person). Sixty-two typical children of 3 years-old completed the first-order false belief task “Maxi and the chocolate,” either with a human or with a robot. Results revealed a significantly higher success rate in the robot condition than in the human condition. Thus, young children seem to fail because of the pragmatic difficulty of the first-order task, which causes a difference of interpretation between the young child and the experimenter.

## 1. Introduction

For almost 40 years, the explicit question in false belief tasks (FBT) of Wimmer and Perner (1983), in which the child must express the false belief of a character on the state of the world, has been the commonly accepted task to study the Theory of Mind (ToM). Understanding the false beliefs of others is of considerable importance for the cognitive and social development of children. It is required to grasp that others have mental states, subjective representations conditioned to specific knowledge and experiences, distinct from ours. Thus, understanding that beliefs can be different from one person to another (Perner, 1991). Sabbagh and Bowman (2018) highlight that explicit FBT are a simple test paradigm perfectly representative of this understanding. In these tasks, children must recognize that someone else will behave in a way that does not correspond to how they understand the state of the world.

**Figure 1:**
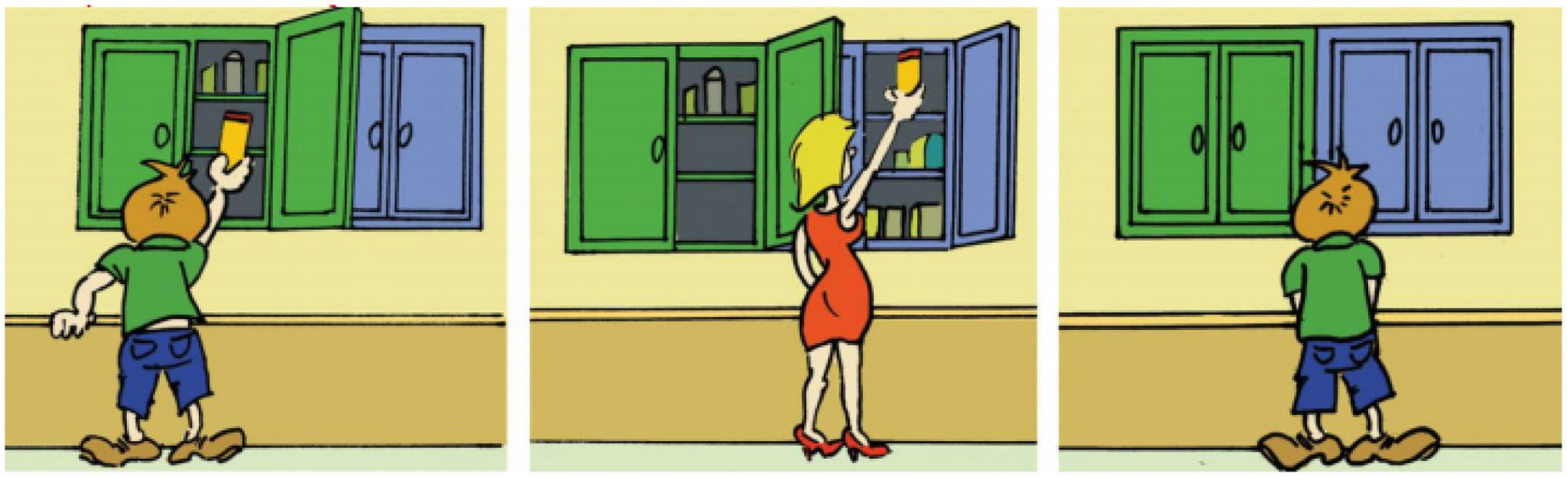
The story “Maxi and Chocolate” of Wimmer and Perner (1983) in clips (taken from Duval et al., 2011, p. 45). Left clip : Maxi comes home from shopping with his mother, and puts the chocolate into the green cupboard before going outside to play. Middle clip: While Maxi is gone, Maxi's mother takes the chocolate from the green cupboard to make a cake and puts it back into the blue cupboard. Right clip: Maxi comes home for a snack. He still remembers where he put the chocolate.

Explicit FBT require a direct verbal answer to an explicit question of the experimenter. The expected answer seems to be very intuitive and is traditionally considered to be a reliable indicator of the understanding of false beliefs. The explicit FBT of Wimmer and Perner (1983) is the following task: The experimenter tells the child participant a story of object transfer through the use of clips^1^: Before going out to play, the child Maxi puts his chocolate into the green cupboard. While he is outside, his mother moves the chocolate and puts it into the blue cupboard. Maxi then comes back to get his chocolate (see Figure 1)^2^.

The child must predict which cupboard Maxi will open to try to get his chocolate. To get the child's answer, the experimenter asks the following test question (ToM question): “Where will Maxi look for the chocolate?” In this question, children are invited to indicate that Maxi will look for the chocolate where he believes it is (i.e., where he left it) instead of where the children know it really is. To answer correctly (green cupboard), the child must activate in their mind the false belief of the character Maxi, who doesn't know the chocolate has been moved, while inhibiting their own knowledge of the world (the chocolate is in the blue cupboard). A control question is then asked by the experimenter following the ToM question to make sure the child understood the story. If the child answers correctly to the ToM question, a “reality question” is then asked regarding the true location of the chocolate at the end of the story: “Where is the chocolate really?” If the child instead fails to answer the ToM question, the next question is then a “memory question” to see if they remember where the chocolate was at the beginning of the story: “Do you remember where Maxi put the chocolate in the beginning?” Results of numerous studies done with neuro-typical children of various cultures (Callaghan et al., 2005) indicate that the majority of 4 years-old children answer the blue cupboard to the ToM question (where the chocolate actually is). It is necessary to wait 4–5 years to see children answering correctly that Maxi will look into the green cupboard (Wimmer and Perner, 1983; Baron-Cohen et al., 1985; Wellman et al., 2001; Sabbagh and Bowman, 2018). The explanation being that children between 3 and 5 learn conceptual knowledge necessary to make explicit decisions about the representative mental state of others^3^.

Yet, these results seem to be in contradiction with behavior observed in 3 years-old children requiring first order abilities, such as the game of Hide and Seek. In this game, the child must go somewhere they will not be seen by others. To succeed the child must understand the difference between their knowledge and what others will perceive. Children younger than 4 are able to evaluate what can be perceived by others, and thus to adopt a point of view different from their own (Shatz et al., 1983; Reddy, 1991, 2007, 2008; Bartsch and Wellman, 1995). The first order ToM then seems to be an ability acquired before the age of 4 (Baillargeon et al., 2010; Westra and Carruthers, 2017). Hala et al. (1991) show that children who failed an explicit FBT, in an ecological situation, can understand and use the false beliefs to explain the mental state of the protagonist of the story. The reasons of the systematic failure of 3 years-old children would be, for these authors, due to the specificity of the explicit FBT. The child must give conscious and declarative answers to the questions of the experimenter. Explicit tasks with verbal answers would require important cognitive resources. These tasks would greatly involve executive functions; such as the ability of the child to inhibit their own point of view to consider that of others. These executive functions would still be immature at the age of 4 (Leslie, 2005; Baillargeon et al., 2010; Westra and Carruthers, 2017; Oktay-Gür et al., 2018, for a discussion). In implicit FBT, in which the answers of children are deduced from actions or gazes and not from explicit pointing or linguistic replies, a much more precocious success (starting from 15 months old) is observed (Onishi and Baillargeon, 2005; Southgate et al., 2007; Surian et al., 2007; Baillargeon et al., 2010; Scott et al., 2010; Heyes, 2014).

In consequence, there is a “developmental paradox of the understanding of false beliefs” (De Bruin and Newen, 2012; Newen and Wolf, in press): toddlers succeed in implicit FBT using behavioral responses but kids below the age of 4 generally fail the explicit FBT in which they must explicitly answer the experimenter's question. Some following a “nativist” approach argue in favor of an early ability to detect false beliefs (based on an innate module) allowing toddlers to succeed in implicit FBT (Leslie et al., 2004). Others following a more “empiricist” approach argue that the ability to understand false beliefs is due to the development of cognitive abilities. It is that development which is responsible for the change of performance in explicit FBT at the age of 4 (see Newen and Wolf, in press, for a recent review). Newen and Wolf (in press) point out a distinction dividing both nativists and empiricists into those who give a cognitive explanation and those who give a situational explanation to the failure of children. For the former, explicit FBT would be difficult because the correct answer would require cognitive resources not yet developed for children between 3 and 4. For the latter, the failure in explicit FBT would actually be the result of the procedure itself; which would be a source of the misunderstanding of the question for these children. Our study focuses on this situational explanation (and in particular the pragmatic explanation) of the failure of toddlers in explicit FBT. We suggest a new procedure able to cancel out situational factors without modifying the structure of explicit FBT themselves. Still, we believe that the situational explanation is profoundly cognitive as well as the Relevance Theory (Sperber and Wilson, 1986) we use to explain the influence of the situation is a fundamentally cognitive theory.

Helming et al. (2014, 2016), Westra (2017), and Westra and Carruthers (2017) all consider the failure of children younger than 4 to be caused by a defective understanding of the expectations of the experimenter in the question. The correct interpretation of the ToM question would require a cognitive effort too great for children of that age. Furthermore, since discussions on beliefs are not common, children would systematically interpret the expectations of the experimenter to be about testing the child's knowledge about the state of the world (i.e., indicating where the chocolate really is) compared to the beliefs of a fictive character. This incorrect interpretation of the ToM question would be caused by the conversational context: the attribution of the status of teacher to the experimenter, and their own status of pupil. We suggest transforming this context (1) By switching the roles and specific statuses of the experimenter and of the child participant and (2) By replacing the experimenter with an “ignorant and slow entity.” This context, we call “mentor-child,” disambiguates the ToM question asked by the ignorant entity by making it clear that it expects to understand the false belief of Maxi. To do this, we replace the experimenter with a humanoid robot NAO. This work will be organized in the following way: After recalling the obligation to consider the pragmatic implicatures in all acts of communication, we will expose those driving the child to produce an incorrect answer in the explicit FBT. We will then explain a new procedure to diminish the ambiguity of the questions. After describing the results, we will discuss them and conclude with the suggestion of future areas of research.

## 2. The Ambiguity of the ToM Question

Sperber and Wilson (1986, 2002) have shown that all communication is inevitably of a pragmatic nature. A communicator performs in a way, such as producing a speech act or a gesture, and the receiving audience must understand the intent hidden beneath the surface. It is especially important to understand that most of the experimental paradigms in cognition, social cognition and developmental cognition correspond to an act of communication between an experimenter and participants. There are many examples in the psychological literature that answers given, considered to be incorrect by the experimenter, by adult participants are actually the result of the participants' misunderstanding of the intentions of the experimenter. The utterances used and the context of the experimental task trigger implicatures in the participants that can induce answers that are different from those expected by the experimenter (see Dulany and Hilton, 1991; Sperber et al., 1995; Baratgin and Noveck, 2000; Macchi, 2000; Politzer and Macchi, 2000; Baratgin, 2002, 2009; Bagassi and Macchi, 2006; Baratgin and Politzer, 2006, 2007, 2010; Macchi and Bagassi, 2012; Macchi et al., 2019, 2020, for examples). Many developmental studies also give pieces of evidence for the ability of children, given their age, to recognize the intentions of the communicator (see Braine and Shanks, 1965a,b; McGarrigle and Donaldson, 1974; Rose and Blank, 1974; Markman and Wachtel, 1988; Politzer, 1993, 2004, 2016; Gelman and Bloom, 2000; Diesendruck and Markson, 2001; Bagassi et al., 2020, for examples).

Sperber (1994) suggests that the child uses the simplest procedure of interpretation which consists in inferring from the communicative stimulus the most relevant intention in relation to their own point of view. However, what is relevant for the child may be different from what the experimenter actually intends to communicate. Thus, by analyzing the experimental task of Piaget and Szeminska (1941) on the class inclusion question, Politzer (1993, 2004, 2016) has shown that the performances of children in relation to their age could be explained by the differences of their interpretation of the question. The experimenter showed five asters and three tulips. The child was then asked whether “there are more asters or more flowers.” The typical answer of children under 8 is “There are more asters.” Politzer demonstrates that the question can be characterized by an ambiguity at the root of the response of the youngest children. While the question of class inclusion is enunciated, according to the relevance principle (Sperber and Wilson, 1986), children will try to infer the expectations of the experimenter and to adapt their answer so that it feels relevant to them. Questions are relevant when they make the person to whom they are asked answer in a relevant way (i.e., questions that require the least cognitive cost for the most contextual effect). These assumptions depend on the representational attributions of the child for the experimenter which are a function of their development (Hayes, 1972). According to Politzer, young children do not make mistakes of class inclusion. They simply have a different representation of the question, making them give an incorrect answer.

“This is a fundamental insight. Once this view is adopted, the disambiguation of the question must be envisaged in relation to the child's development. From the notion that the children attempt to render the question optimally relevant it follows that the way they do so will vary with their cognitive development. In other words, the interpretation chosen by the children is constrained by their level of development. Therefore, the interpretation can be predicted based on what is likely to be the children's estimation of the relevance of the question” (Politzer, 2016, p. 3).

Politzer observed that when he disambiguated the question of class inclusion the success of participants was significantly improved and came earlier: between 5 and 6 years-old (see also Jamet et al., 2018).

It is then legitimate to wonder if, like with the question of class inclusion, the incorrect reply given by young children in explicit FBT could also be the result of a different interpretation of the ToM question which would be caused by an incorrect inference of the experimenter's expectations. With the years, and with the acquisition of the pragmatic skills of the child, the ambiguity of the question would later decrease. This pragmatic hypothesis could explain the early success in implicit FBT, which are simplifications of explicit FBT in which the ToM question is not explicitly asked. To succeed in these tests, the child does not need to correctly interpret the question or to correctly infer the intention of the experimenter. They only need to understand the false beliefs. For Siegal and Beattie (1991); Westra (2017); Westra and Carruthers (2017), since the beginning of their development, young children can create representations of others' beliefs and understand the false beliefs. However, 3 years-old children do not expect beliefs to be a likely topic of conversation (Westra, 2017). It is difficult for them to induce that facts relative to someone's beliefs can be a relevant topic in the conversation with the experimenter and that this is what the question is about. Despite the fact that young children constantly attribute propositional attitudes to other agents, understanding when these pre-linguistic concepts play a part in the conversation is not only a question of acquisition of the adequate vocabulary but would also be a question of the development of pragmatic skills (Westra and Carruthers, 2017). The child must be exposed to conversations for these social stimuli to play a crucial part in the strengthening of their linguistic and pragmatic skills (Astington and Olson, 1995; Carpendale and Lewis, 2004; Antonietti et al., 2006; Westra, 2017)^4^.

This lack of pragmatic skill is even more salient in explicit FBT as the conversational interaction happens between the child and a stranger (the experimenter). At the age of 3, even if the young child has had numerous interactions with their parents and family, interactions with adults are generally limited, except for the teacher which is for most still a recent interaction (3 is usually the age at which children start school). The teacher is certainly an important reference for the young child during the experiment. After 2 months of class, preschool children have integrated the didactic contract wanted by the teacher. The teacher explicitly invites the pupils to work well, to show everything they know. Each time the child returns to class, after completing an activity, the teacher will ask them if they worked well. As Westra and Carruthers (2017) explained, children are readily able to consider that the interaction with the experimenter has an educational intention. Indeed, educational clues are almost always present in an explicit FBT. The experimenter, for the child, is in a social position much superior to theirs and, just like their teacher, has the encyclopedic knowledge. The experimenter-child relationship reinforces this impression of superiority since the experimenter is introduced to the child as an authority figure to whom they must obey. This attribution of teacher is facilitated even more by the fact that the experiments most often happen at school, during school time. This supposition of an educational intention in the task implies, for the child, that an educational behavior is expected of them, as it is usually the case in this context. Therefore, they are in a position of pupil during the experiment. How uncommon the situation is, an adult replacing the teacher for an educational exercise, can strengthen the idea that this exercise is really important and that this new teacher may be special and knows more than the usual teacher. This attribution is all the easier since the experimenter is often presented as a researcher, a specialist. Preschool children indeed seem to be already sensitive to the knowledge of the informant in educational activities (Jeong and Frye, 2018b).

When teaching a new concept in an example or a story, the teacher later checks the child understood correctly through simple and direct questions linked to what was just told. These questions are very rarely ambiguous. The correct answer expected by the teacher is usually meant to prove that they understood the story correctly. Thus, to the child, the same can be expected of the questions asked by the experimenter. The main difficulty in explicit FBT is the fact that they involve four different elements of knowledge: (1) Where Maxi initially put the chocolate, (2) The change of location done by Maxi's mother, (3) The fact that this change of location happened in Maxi's absence, and (4) The fact that Maxi is looking for his chocolate, probably in the wrong place. For the child, there are multiple possible interpretations of the experimenter's expectations when they ask the ToM question. They can be: trying to assess whether the child understood the change of location of the chocolate (steps 1 and 2), or assess whether the child understood the fact that Maxi was not there during the change of location, and that in consequence he will look for the chocolate in the wrong place: the initial location (steps 3 and 4). Along these interpretations, the one which concerns the attribution of beliefs to someone else has a greater cognitive cost for young children. They are generally not experienced enough in interacting with adults to grasp the relevance of this expectation. Children of 3 years-old will instead use the more familiar interpretation: they will think that the experimenter expects the reply to be about the child's understanding of the change of location (Siegal and Beattie, 1991; Hansen, 2010; Lewis et al., 2012; Westra, 2017).

Helming et al. (2014, 2016) offer a more elaborate pragmatic explanation of young children's answer. For them, explicit FBT force the children to adopt two points of views at the same time. One is more detached: “spectating” in the third person the action of the main character of the story, in particular focusing on the character's beliefs; and the other is more communicative: interacting with the experimenter in the second person. This first point of view being disrupted by the second. The ToM question then generates two biases: one “referential” and one “cooperative.” Children have the possibility of mentally representing the real location of the chocolate or where Maxi wrongly believes it is. Using the word chocolate in the question can bias children toward answering with the real location (referential bias). The interaction with the experimenter would bring the child to focus on the knowledge they share (i.e., the real location of the item). This would then disrupt the ability of the child to track the false belief of the main character from the third person point of view. In essence: when the experimenter refers to the target item, they direct the attention toward the real location. The cooperative bias is the result of the tendency of toddlers to want to make themselves useful by spontaneously helping others (even adult strangers) to reach their goals, even if it requires a greater effort and if they are busy with a task of their own (see Warneken and Tomasello, 2007, 2009, 2013; Liszkowski et al., 2008; Buttelmann et al., 2009, 2014; Warneken, 2015). This helpfulness seems to be mainly motivated by an intrinsic care for the other and not for any personal reward (Hepach et al., 2012, 2016). This tendency to help others made it possible to create implicit FBT. The task given to toddlers consisted in helping an adult reach their goal. Yet to infer this goal, the toddlers needed to consider what the adult believed. This tendency would drive children to adopt a second person point of view toward the main character of the story, rather than a spectating point of view in the third person, this in turn driving them to incorrectly interpret the expectations of the experimenter. Children understand that the main character needs help, because he has false beliefs, to avoid picking the wrong location. They spontaneously want to help him by telling him the correct location and can readily expect to be invited to do so. This, for the child, would strengthen the interpretation of the ToM question “Where will Maxi look for his chocolate?” as an invitation to help the main character find the item. This means interpreting the question as a normative question “Where should he look for his chocolate?” or even “Can you tell Maxi where to find his chocolate?” As Newen and Wolf (in press) point out, this pragmatic explanation is not in contradiction with the cognitive explanation (in terms of “mental files” by Recanati, 2012) suggested by Perner et al. (2015), Perner and Leahy (2016), and Huemer et al. (2018). These mental files, or mental representations, include the “information management tools about an object in the world” and the links between the different files which make it possible to share information between them. In “Maxi and the chocolate,” the child has two mental files of the situation: one “regular” file with the information that the chocolate is in the blue cupboard, and one “indirect by proxy” file indexed on Maxi with the information that the chocolate is in the green cupboard. According to Perner and Leahy (2016), when children below the age of 4 are faced with the ToM question, they are not yet able to switch between the indirect mental file and the regular mental file in a controlled and systematic way. It is only once the mental files are linked that the child can access the information about Maxi's beliefs. The pragmatic explanation, through the Relevance Theory, allows us to understand which mental file will be activated. In a traditional context, the mental file which has the least cognitive cost and the greatest contextual effect is the regular mental file which answers what the child believes to be the experimenter's expectation.

Thus, as Westra and Carruthers (2017) pointed out, there are two interpretations at stake in addition to the correct interpretation of the ToM question for a total of three possible interpretations: (1) The “helpfulness-interpretation” where the question corresponds to an invitation to help the character, (2) The “knowledge-exhibiting-interpretation” where the question corresponds to an invitation to show one's knowledge of the events in the story (steps 1 and 2 as described in the previous paragraphs), and (3) The “psychological knowledge-exhibiting-interpretation” where the question corresponds to an invitation to report the character's false beliefs about the location of the object (steps 3 and 4)^5^. The child's task is to determine which of these three competing interpretations is most likely to meet the experimenter's. Interpretation (3) is the one expected by the experimenter. Each of the other two leads to the incorrect answer of indicating the actual location of the chocolate. As indicated above toddlers do not yet have the pragmatic experience required to understand that people's beliefs are a valid topic of conversation. In consequence, they are more inclined to interpret the ToM question as a kind of indirect language act to verify their knowledge of the real location of the chocolate (interpretation 1). This will also help the character find the chocolate (interpretation 2). As children gain experience in discourse about the beliefs of others, they begin to be able to recognize the true purpose of the question and their true expectation (interpretation 3): reporting explicitly the false belief of the character called Maxi (Westra and Carruthers, 2017; Frank, 2018). They then understand that the question “Where will Maxi look for the chocolate?” implicitly means “What does Maxi falsely think about the location of the chocolate?”

A number of authors have tried to directly disambiguate the ToM question. Siegal and Beattie (1991) give the following question [reformulated to fit Maxi and the Chocolate]: “Where will Maxi look for the chocolate first?” which directly explains the experimenter's expectation. The authors observe a significant increase in correct answers (Yazdi et al., 2006; Białecka-Pikul et al., 2019). Hansen (2010) also observes much better results when the experimenter directly specifies in their question that they are not interested in the child's knowledge of the state of the world [reformulated to fit Maxi and the Chocolate]: “You and I know where Maxi's chocolate is, but where does he think it is?”

Another solution is to explicitly and conceptually explain the important clues in the story to make the correct interpretation (3) of the ToM question more conceptually relevant (Newen and Wolf, in press), for example by making the false belief of the main character more salient. Mitchell and Lacohée (1991) noticed that children participating in explicit FBT who kept an explicit aide-memoire of their prior belief (the cupboard where the chocolate was [reformulated to fit “Maxi and the Chocolate”]) was much more successful at avoiding a later deformation of this belief. Lewis et al. (2012) showed that the explanation of the false beliefs of another person is improved if we add another character to the story who is also observing object's change of location. The presence of the other person conceptually highlights the possible point of views in the story. In this situation the ToM question, being explicitly directed at the character who did not see the change of location, increases the relevance of interpretation (3) on the false beliefs of the character. Rubio-Fernández and Geurts (2013, 2016) demonstrated that toddlers can also succeed in explicit FBT if the task is modified in such a way that, first, the point of view of the other person is frequently repeated to the child during the experiment and, second, the ToM question asked to the child is transformed into “What happens next?” Here the disruption induced by the experimenter focusing on the item is no longer possible. It is also possible to make interpretation (2), of exposing the child's knowledge about the real location of the item, less contextually relevant. Wellman and Bartsch (1988), Mascaro and Morin (2015), and Mascaro et al. (2017) indeed notice better performances when the children themselves do not know where the actual location of the item is or if the item is removed from the scene.

Finally, it is possible to change the experimental procedure to make the spontaneous tendency of children to be useful, which usually drives children toward the “helpfulness-interpretation” (1), to become an indicator of the effective false belief of the character. Matsui and Miura (2008) showed that toddlers succeeded more easily when the task was changed to have them choose a character whom they had to help find the item (pro-social context).

To sum up: whether children can disambiguate the ToM question depends on their meta-cognitive development. Toddlers make the question more relevant by interpreting it as a question about their knowledge of the story or, with the same result, a question about their knowledge of the story to help the main character. Older children interpret it correctly to be a request for them to report their knowledge of the false belief of the character in the story.

## 3. Changing the Context to Disambiguate the ToM Question

In all these experiments the original task is modified. The ToM question is sometimes modified, the participant is sometimes asked to keep in memory the initial belief, a character is sometimes added or some information is sometimes removed. Our objective is to decrease the salience of incorrect interpretations without changing neither the story nor the question asked: by playing with the global context of the experiment itself. A good example is the length and number conservation task (Piaget and Szeminska, 1941). Assessing the conservation of number is done by presenting two lines of tokens, equal in number and arranged in a one-to-one correspondence, in front of a child who judges them to be the same. When the experimenter rearranges one of the rows the non-conserving child changes their judgment in favor of the longer row. McGarrigle and Donaldson (1974) showed that when the transformation of the row of tokens is the indirect result of an action with a different goal, such as a transformation effected by a “naughty teddy bear” who wants to “spoil the game,” children are more conservant. In this “accidental transformation,” there are no structural modification of the task.

As explained above, the way the child interprets the questions of the experimenter in explicit FBT depends, in part, on their understanding of the nature of the communicative exchange (i.e., its topic and goal). For toddlers, the context of the task, as shown above, strongly expresses that of a school activity with the status of the experimenter-teacher, able to judge, and the location. Thus, the child infers effortlessly their role in this task will be the one they already know and are used to during classes: that of a pupil with the goal of learning and show their knowledge. These assumptions made by the child for the experimenter to be testing their knowledge are the origin of interpretations (2) and (3). The “helpfulness-interpretation” (1) can be considered to be the desired expectation in order to help the character in the story (numerous studies cited above indicate how spontaneously, and without ulterior motives, the toddler displays an altruistic behavior). Thus, if we had an experimental context in which exposing the knowledge of the false beliefs of the character (interpretation 3) could also satisfy a “helpfulness-interpretation” (interpretation 1), then interpretation (2), about the actual state of the world described in the story, could be inhibited.

### 3.1. A Mentor-Child and an Ignorant, Naive, and Slow Pupil

To do this we must consider a situation which would change the assumptions of the child about the person asking the ToM question; a situation in which the child could spontaneously infer that an answer indicating the false belief of the character would help the person asking this question^6^. A first modification of the context would then have the person asking the ToM question display an explicit need to know the false belief of the character. The person would have trouble understanding the story, as for them the answer to the ToM question is far from obvious, even if they asked it. This person must have less knowledge than the child and must consider the child to be someone who knows more. Thus, we must consider a context in which the status of the child and of the experimenter are switched compared to the original context.

We can imagine a “mentor-child” context in which the young child must answer the questions of an ignorant entity introduced by an authority figure: “You are the teacher and this is your pupil. It doesn't know much. It needs you^7^.” In the conversational act, the expectation of the child regarding the questions of the entity is to be able to help it learn new things. Let us imagine that this ignorant being tells the child: “I was told a story that I didn't understand very well. I'll tell it to you and then please explain it to me.” After telling the story, the ignorant entity asks the ToM question in a naive tone. The child answering correctly shows their knowledge while helping the entity. The question here is disambiguated and reliably drives the child toward interpretation (3). The question asked in this context becomes natural, for the child knows the entity to be ignorant and that it can ask trivial questions. This is not the case in the traditional context where it can seem surprising that a “knowing” adult could ask such a question.

Another important aspect is to highlight the “naive,” “unsure of itself,” and “slow” traits of the ignorant entity. This aspect helps the child consider themselves knowledgeable compared to it. It also helps the child feel useful when helping it. More importantly, the “slow” aspect of the entity can favor the interpretation of the control questions asked depending on the success or failure in the ToM question (respectively the reality question and the memory question). To our knowledge, the pragmatic analysis of these questions has never been explicitly done in the literature. This can certainly be explained by the fact that in Wimmer and Perner (1983), all children succeeding in the ToM question also correctly answered the reality question^8^. In the standard context, the interpretation of the reality question is indeed completely obvious for the child. It corresponds to the question that is the most expected; which has the strongest contextual effect and requires the least cognitive cost to answer it. After indicating the false belief of the character in the ToM question, the reality question makes it even more explicit by indicating where the item actually is. This second question does not seem to be incongruous in the standard context in which the child assigns the status of teacher to the experimenter. A teacher often asks multiple questions to test the knowledge of the child. In our “mentor-child” context, the reality question asked by the ignorant entity can seem to be a bit odd to the child in their role of teacher. Indeed, asking this second question requires understanding that the chocolate is in a different place than where the character believes it is^9^. Thus, the ignorant entity, if it did understand correctly the answer given by the “mentor-child” to the ToM question, should have also understood that Maxi has a false belief and will look into the green cupboard which is now empty. Frequently, when a pupil asks a second question to the teacher just after receiving an answer to another, it is often because they need more precision or because they did not understand the answer. In this case, there are two possible answers for the “mentor-child”: (1) Thinking that they were not clear enough with their first answer and be inclined to repeat the same answer as in the ToM question, or (2) Accurately answer the reality question to give some new information to help the entity understand their first answer to the ToM question. In order to increase the chances of this second option, the entity must not simply be perceived by the “mentor-child” as “ignorant” but also “a bit slow.”

In a similar fashion, the memory question, asked after an incorrect reply to the ToM question, can also be interpreted as a request for confirmation of the understanding of the first answer. Yet, in the standard context, the memory question can seem to be disrupting for children younger than 4 incorrectly answering the ToM question as the final location of the item is given at the end of the story. Indeed, this answer implies having followed the change of location of the chocolate during the story and to remember the initial location of the chocolate. The weak performances observed (37.8% of success in the memory question) in Wimmer and Perner (1983) for children between 3 and 4 may not be the result of the difficulty of the task but instead be the result of the ambiguity of the question for their age. Older children, because of their conversational experience, may more readily reinterpret the memory question to be controlling their initial answer to the ToM question^10^.

### 3.2. The Robot-Pupil Solution

There is an important literature showing the advantages of using a humanoid NAO robot in social interactions with young children, especially in situations of *learning by teaching* (see Jamet et al., 2018). Studies have shown that in conversational interaction with an artificial agent, even completely virtual ones, humans automatically detect pragmatic violations of their speaker (Jacquet et al., 2018; Jacquet et al., 2019a,b,c; Lockshin and Williams, 2020). It was shown that children as young as two can be susceptible to the conversational violations of a robot (Ferrier et al., 2000). Recent studies (Yasumatsu et al., 2017; Martin et al., 2020a,b) also showed that the natural and spontaneous propensity of young children to try being useful extends to humanoid robots seeming to be in difficulty. It seems that 3 years-old children assign mental states to a robot (Di Dio et al., 2018, 2020a; Marchetti et al., 2018). Di Dio et al. (2020a) observed in 3 years-old children who had already developed a first-order ToM skill a tendency to represent the emotional state of a robot in terms of mental states. For these authors, there could be an attempt to anthropomorphize the robot on the emotional dimension which, at the age of 3, could be particularly salient. This suggests that young children are eager to think about the robot mind in the same way they do about the human mind (Di Dio et al., 2018). The NAO robot was also used to study the endowment effect in adults (Masson et al., 2015, 2016; Masson et al., 2017a,b).

The effectiveness of our “mentor-child” context^11^ was tested with children between 5 and 6 in the class inclusion task and successfully made the question of class inclusion more relevant for the child when it was asked (Masson et al., 2017a; Jamet et al., 2018). We hypothesize that the “mentor-child” context should similarly decrease the ambiguity of the ToM question to make it clearer that it is a request about the mental states of the character Maxi. The performance of preschool children should then be significantly improved without changing the original explicit FBT. Should this be observed, we would conclude that the understanding of false beliefs develops before the age of 4 and that the abilities of young children are underestimated due to pragmatic factors.

We also believe that the “mentor-child” context can keep the control questions unambiguous. Therefore, we expect to have a rate of correct responses to the control questions that should be roughly equivalent to that of older children in the standard context.

## 4. Experiment: Explicit False Belief Task in the Mentor-Child Context

### 4.1. Materials and Methods

#### 4.1.1. Participants

We recruited 62 native French children in preschool at “Les Petits Princes” in Versailles, France. The sample chosen in the classes was composed of 34 girls and 28 boys, from 38 months-old (3 years and 2 months) to 49 months-old (4 years and 1 month)^12^. The mean age of children was 44 months-old (*N* = 62, *M* = 44 months-old, *SD* = 2.82 months-old)^13^. The children were randomly assigned a condition depending on their age and gender. These conditions were “human experimenter” (“human” condition) and “robot experimenter” (“robot” condition). Each condition contained 31 children between 38 months-old and 49 months-old (*N* = 31, *M* = 44 months-old, *SD* = 3.47 months-old for the “human” condition and *N* = 31, *M* = 44 months-old, *SD* = 3.09 months-old for the “robot” condition).

#### 4.1.2. Materials

The story “Maxi and the chocolate” was shown to the child with the clips displayed in Figure 1. Each clip was 6.4 × 5.8 cm (2.5 × 2.3 in). The clips were shown in a black and opaque folder containing a cardboard spacer in A4 format (21 × 29.7 cm or 8.3 × 11.7 in). All three clips of the task were attached to the cardboard spacer in advance. The robot used in this experiment was a 58 cm tall (23 in) NAO robot created by Aldebaran Robotics (Aldebaran version 4—“Evolution”). It has a moving head, arms and hands, each with three fingers, allowing it to point at the clips of the story to punctuate its discourse with gestures. NAO is also equipped with a microphone and speakers to communicate with humans. The robot was remotely controlled by the experimenter using a computer, but its gestures and speech were recorded in advance. They could see the child thanks to a camera in the eyes of the robot. The movements and the speech sections triggered in real time avoided having too much variability between the different participants, while still making it possible to make the answers of the robot fit those of the child. We chose to remotely control the robot for logistical reasons: even though NAO does have the ability to recognize speech making it possible for it to autonomously react, the behaviors of children can sometimes be unpredictable. Some flexibility was needed to reproduce with fluidity a natural conversation with a human. Moreover, children could sometimes speak too low to be understood by the robot, which would have made the interaction impossible. Finally, the robot also allowed a better standardization of the enunciation context thanks to its intonations and utterances being strictly identical across all participants. To make NAO more childish and less intimidating, its voice was manipulated so that it had a higher pitch and spoke more slowly. NAO was programmed to blink randomly during the experiment to strengthen its humanness.

#### 4.1.3. Procedure

Before the beginning of the experiment one member of the research team, that we call the companion, was welcomed into the class and gave their name. Children were sitting in a circle in front of the teacher. She explained that this new person was there to make all the children of the class work on a task, a bit like teacher. The procedure in both conditions was subdivided into two sequential steps: the priming step and the explicit FBT.

##### 4.1.3.1. Human Condition

In this condition the companion told the children they would be participating in an activity if they agreed. After this introduction each child was guided to the location of the experiment, in a quiet multi-purpose room of the school. During the walk, the companion told the child the didactic contract: “You're about to listen to a story, like in class, and my colleague [name of the experimenter] will ask you some questions. You will need to answer them.” The companion then asked for the agreement of the child. If the child agreed, the child then entered the room without the companion and stayed with the experimenter.

The experimenter then introduced themselves to the child who was seated on a chair in front them. The child's ability to correctly name the two colors (blue and green) was checked before the main task^14^. The false belief story was then verbally told and illustrated with clips, which allowed non-verbal answers for children which preferred to point at their answer instead of saying them.

If the child's answer to the ToM question was the green cupboard, the experimenter pointed at it on the clip and said “ah it is there.” If the child did not change their initial answer, the answer was considered to be correct. When the child instead gave the incorrect answer, no confirmation was required, and the answer was immediately considered to be false.

The reality question and the memory question were then asked (respectively following the success and failure to the ToM question).

Finally, the experimenter thanked the child, and the companion guided them back to the classroom while congratulating them.

##### 4.1.3.2. Robot Condition

In this condition, the companion explained that they came with a NAO robot. They told the class NAO needed the children's help because it knew nothing while they all knew a lot. If one child doubted of their knowledge, the companion told them that they were learning many things in class but also that they already knew a lot. More importantly: they knew more than the robot. The companion then asked if the children agreed to teach things to NAO^15^.

Like in the “Human” condition, the companion guided each child individually from the class to the location of the experiment and told them the didactic contract: “Your job is to teach lots of new things to NAO. NAO is a little robot who knows nothing. NAO needs you to learn new things. NAO doesn't know anything. You will be his teacher. Do you agree to be his teacher?” To make the child understand NAO's ignorance the companion pointed at the child's clothes, or various items in the location of the experiment. They asked the child to name them, which was done without any difficulty, and then they told the child:

“You see, NAO doesn't know all that. If NAO asks you weird, strange questions, you must answer him. Remember that he knows nothing. If NAO tells you strange things, or if he makes mistakes, you correct him^16^. You are his teacher. Do you agree to be NAO's teacher [name of the child]?”

**Figure 2:**
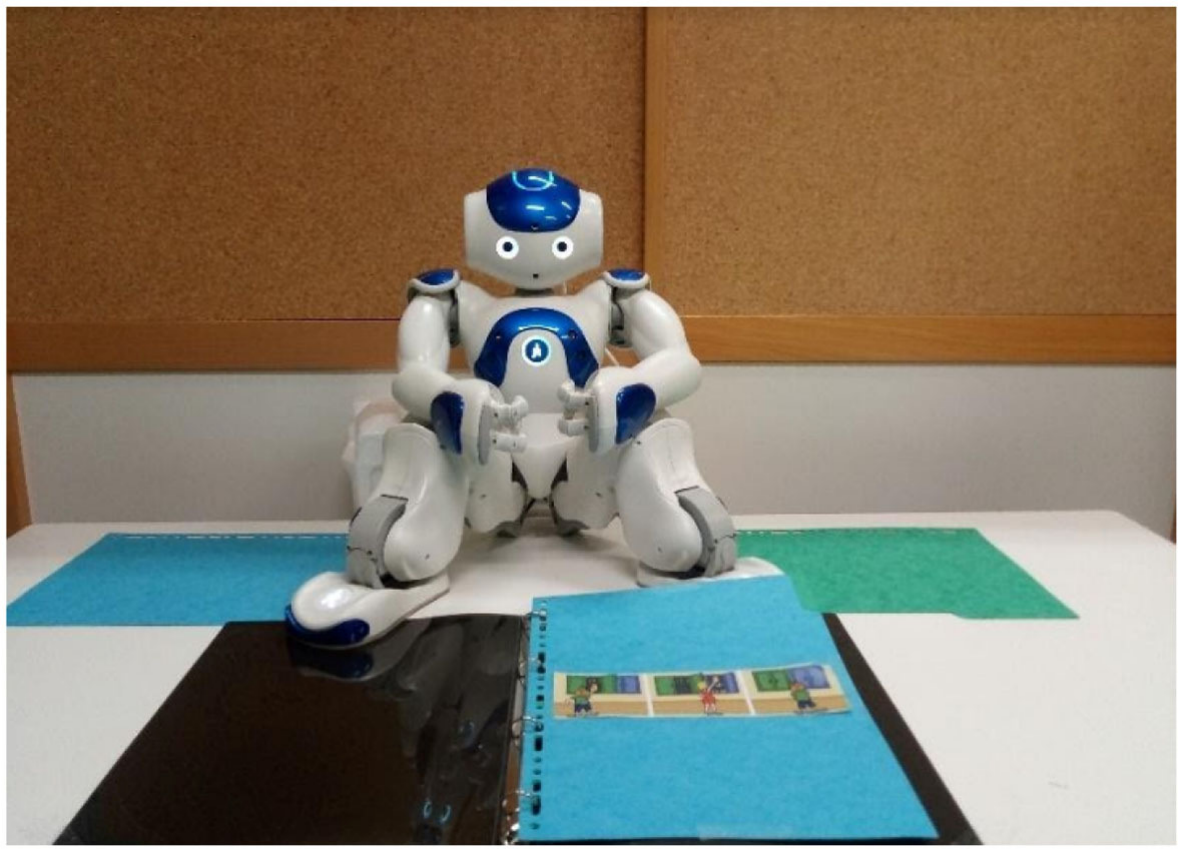
Robot experimenter and materials for the “Robot” condition. The NAO robot is seated on a table in front of the child.

If the child agreed, the companion let the child enter the experiment room and left the child “alone” with the robot (see Figure 2). This is an especially important detail with the robot. Indeed, should the companion remain in the room, the child may be tempted to answer the robot in the same way they would with a human experimenter because of the presence of an adult in the room. Pragmatic interpretations would then be modified. The actual experimenter was hidden behind a screen, without the child knowing about it, and remotely controlled NAO using a laptop.

NAO introduced itself to the child. It asked if the child agreed to be its teacher because he was there to “learn many things.” Once again, if the child did not agree, the experiment stopped. The robot then asked the child if they can help it learn colors. NAO then pointed the colored cardboard sheets and made mistakes (for example: NAO said “That's yellow?” while designating the blue cardboard, making it more believable the fact that NAO did not know much and thus strengthening the role of the child as a teacher). To further strengthen the naive aspect of the robot, NAO insisted on its ignorance all along the experiment (e.g., “Alright, I had not understood that. I am really stupid.” It is important to note that great care was given to not overdoing the “stupidity” of the robot. Indeed, if its mistakes became too predictable, there was a great risk of losing the child's interest in teaching it anything. A child could quickly have inferred that “NAO will make mistakes no matter what I tell him.” which could have biased the child's experience in the task if it had not been controlled.

NAO initiated the story “Maxi and the Chocolate” by telling the child “A man told me a story that I did not understand. Do you want to help me?” Identically to the “Human” condition, NAO told the story and then asked the ToM question and the control question.

The answer to the ToM question was considered to be correct if the robot was corrected by the child when it made a mistake trying to repeat the answer. For example, if the child answered that Maxi will look for the chocolate in the green cupboard (initial position), NAO said: “Ah thanks, so if I understood well the chocolate is in the blue cupboard.” If the child corrected NAO and said: “No, the chocolate is there.” (indicating the green cupboard) or “No, it is in the green one.” the answer was considered to be correct. Note that, just like in the human condition, the child could also point at the clips directly instead of answering verbally.

Once the task was over, NAO thanked the child for being its teacher “Thank you, you've been an awesome teacher. I've learned many things thanks to you!” and told them goodbye. The companion then came to bring the child back to the classroom. Sometimes the teacher asked how the task went. The companion congratulated the child for the quality of their teaching. They told the rest of the class that NAO still needed to work to learn things. This way, the fact that NAO needed help was progressively very well-communicated to the whole class while they did their usual class activities.

#### 4.2. Results

In this experiment, the dependent variable was a dichotomous variable which modalities were interpreted in terms of *success* or *failure*. According to the procedure used by Wellman and Liu (2004), the child's response was a *success* when they produced correct answers to both the ToM question and the reality question. This variable will be noted below: *TR* (ToM and Reality). We also analyzed the data from a less conservative perspective (Wimmer and Perner, 1983) by interpreting the *success* as being simply a correct answer to the ToM question. This second version of the dependent variable will be symbolized by the letter *T*. Finally, we also analyzed the answers to the memory question for children who had failed to answer the ToM question correctly. This variable will be designated by ¬*TM* (Not ToM and Memory)^17^.

The independent variable (noted *C*) had two modalities: “Human” vs. “Robot.” We also tested the influence of two other variables: the sex of the child (noted *S*, with two modalities: Girls vs. Boys), and the age of the child in months (noted *A*, numerical variable ranging from 38 to 49 months-old). In the first step of the analysis, we adjusted a linear model on our data with a link *logit* function and a *binomial* distribution of the errors. We applied this treatment to all three versions of the dependent variable *TR*, *T* and ¬*TM*. For all of them we included, in the linear predictor of the models, the main effects of each of the three factors *C*, *S*, and *A*, as well as all the possible interactions which includes the triple interaction. We refer to these *saturated* models by using the following expressions: firstly *TR* ↫ *C* * *S* * *A*, secondly *T* ↫ *C* * *S* * *A* and thirdly ¬*TM* ↫ *C* * *S* * *A*. The “↫” symbol refers to the influence, supposed or real, of the independent variables on the dependent variable while the “*” symbol indicates that all the possible interactions between the independent variables are taken into account. We then used a procedure of automatic *backward* simplification on all the saturated models to lead to the corresponding final models.

The principal characteristics of these four models are shown in Table 1. Results show, regardless of the version of the dependent variable (*TR*, *T*, and ¬*TM*), that the simplification model systematically terminated on a final model containing only the *C* factor. This means that the success rate for ToM, defined either as the conservative model (*TR*) or as a more permissive model (*T*), remained completely explainable by the condition (i.e., “Human” vs. “Robot”). The same was also observed for the final model of the memory question (¬*TM*). Therefore, in our study neither the sex (*S*) nor the age (*A*) of the children can significantly improve the prediction of the success we observed. Thus, in the rest of the paper these two variables will be omitted.

**Table 1:** Main characteristics of the data-adjusted models.

**Models**	**Residual deviance**	**ddl**	**AIC**
*TR* ↫ *C* * *S* * *A* (saturated)	69.86	54	85.86
*TR* ↫ *C* (final)	73.15	60	77.15
*T* ↫ *C* * *S* * *A* (saturated)	73.37	54	89.37
*T* ↫ *C* (final)	77.57	60	81.57
¬*TM* ↫ *C* * *S* * *A* (saturated)	40.06	28	56.06
¬*TM* ↫ *C* (final)	43.00	34	47.00

*For the different versions of the dependant variable (TR, T, and ¬TM), the simplification of the saturated models led to final models containing only the predictive variable C (the influence of the experimental conditions). In all situations, the resulting models are more parsimonious with a reduction of the AIC (Akaike Information Criterion) of about 8 points*.

A summary of the data we collected is shown in Table 2.

**Table 2:** Distribution of the children's answers depending on the experimental condition (*N* = 62).

**Conditions (C) Questions**	**“Human”**	**“Robot”**	**Total**	**“Human”**	**“Robot”**	**Total**
ToM: “Where will Maxi look for the chocolate?”	“Green cupboard” (*T*)			“Blue cupboard” (¬*T*)		
	8 (26%)	18 (58%)	26 (42%)	23 (74%)	13 (42%)	36 (58%)
Reality: “Where is the chocolate really?”	“Blue cupboard” (*TR*)					
	6 (19%)	14 (45%)	20 (32%)			
Memory: “Do you remember where Maxi put the chocolate in the beginning?”				“Green cupboard” (¬*TM*)		
				12 (39%)	11 (35%)	23 (35%)
Success rate for the control question depending on the ToM answer	(*R*/*T*)			(*M*/¬*T*)		
	75%	78%	77%	52%	85%	63%

*TR (correct joint answer to both the ToM & reality questions), T (correct answer to the ToM question), and ¬TM (correct answer to the memory question for those who only gave an incorrect answer to the ToM question). The breakdown does not take into account the variables sex (S) and age (A) of children because, as the regression analyses have shown, neither of these two variables affects the probability of success to the ToM question. N_Robot_ = 31, N_Human_ = 31*.

Table 3 shows the coefficients associated with each condition for the three resulting models required to estimate the effect size of the condition (*C*).

**Table 3:** Estimated β coefficients associated with belonging to the conditions for the three models.

**Models**	**β (OR)**	***SD* (β)**	***z*-Value**	***p*-Value**
*TR* ↫ *C*	1.23 (3.43)	0.58	2.12	0.03^*^
*T* ↫ *C*	1.38 (3.98)	0.55	2.52	0.01^*^
¬*TM* ↫ *C*	1.62 (5.04)	0.87	1.85	0.06

*The Odds Ratio corresponding to each of the coefficients are shown between parentheses. Since the link function of the models is a logit, calculating the exponential of the coefficients is the only thing required to get the OR*.

* *p ≤ 0.05*.

The model *TR* ↫ *C* has a significant coefficient (β = 1.23, *p* < 0.05). This coefficient is also significant for the model *T* ↫ *C* (β = 1.38, *p* < 0.05). We show in Table 3 the odds ratio (OR) corresponding to the β coefficients. We obtained *OR* = 3.43 for the model *TR* ↫ *C*, which means that the chances of success for a child in the “Robot” condition are almost 3.5 times greater than that of children in the “Human” condition. Regarding the model *T* ↫ *C*, we obtained *OR* = 3.98 indicating that, when simplifying the success criterion, a child was four times more likely to succeed in the “Robot” condition than one in the “Human” condition. We also observed a tendency for children who failed the ToM question to answer the memory question with more success when it was asked by the robot (β = 1.62, *p* = 0.06). While not significant we can still point out that children were five times more likely to correctly answer with the robot than they were with the human (*OR* = 5.04).

Table 2 shows the distribution of the participants depending on the condition and on whether they succeeded in the task (depending on the criterion used to define success). An unilateral proportion test with no continuity correction reveals that the success rate for *TR* is significantly different from chance (χ^2^ = 2.77, *df* = 1, *p* < 0.05). When only looking at the ToM question (*T*) the test does not show a significant difference (χ^2^ = 0.4, *df* = 1, *p* > 0.05). However, as explained above, an answer scored as “correct” for the ToM question needed to be confirmed. Thus, we can probably think that the 58% of children in the “Robot” condition did not simply give the correct answer at random. Only 2 children changed their choices for ToM question in the “Robot” condition (and were counted as a wrong answer for ToM) and none in the “Human” condition.

## 5. Discussion

The goal of this study was to propose a new methodology of the explicit FBT. With it, we hoped to inhibit the erroneous interpretations made by 3 years-old children regarding the ToM question. Our “mentor-child” context seems to have changed the prevailing interpretation of the ToM question in the way we hoped: a request to report the false belief of the character. The young children who participated in our study did better in the “Robot” condition than those in the “Human” condition. This result has three important consequences:

1. It provides new experimental arguments for a pragmatic explanation of the failure of young children in explicit FBT (Cummings, 2013; Helming et al., 2014, 2016; Westra, 2017; Westra and Carruthers, 2017; Frank, 2018).
2. It indicates that a significant proportion of pre-school children can correctly answer the original ToM question.
3. This result, following those of Jamet et al. (2018) on Piaget's class inclusion task^18^, supports the relevance of our methodology to disambiguate the experimenter's expectations through their question in developmental tasks.

In our study, the performance of children in the conjunction of the ToM and reality questions was significantly improved, with children in the “Robot” condition being about 3.5 times more likely to succeed than those in the “Human” condition. Moreover, in the “Human” condition the performances were comparable to those observed in the literature (Wimmer and Perner, 1983; Hogrefe et al., 1986; Perner et al., 1987). The previous result is also amplified if, as Wimmer and Perner (1983), one adopts a laxer interpretation of the success. Indeed, looking only at the recorded responses to the ToM question, our results show that children belonging to the “Robot” condition are 4 times more likely to succeed. Furthermore, although we focused on the performance of pre-school children with participants between 3 and 4 years-old, it is interesting to note that the success rate in our “Robot” condition (58%) is similar to what Wimmer and Perner (1983) considers to be a successful completion of the task for children between 4 and 6 years-old (57%). However, this proportion remains lower than the one recorded by the same authors for children between 6 and 9 years-old (89%). We can point out that this success rate is not as sensational as those observed in some studies (such as the 90–100% observed in Rubio-Fernández and Geurts, 2013, 2016). To our knowledge, we are the first to find such a performance without any modification being made to the initial paradigm with the same scenario, the same questions and the same procedure for analyzing the answers given by the child. Besides, the experimental protocol also considers several methodological criticisms made in the studies cited above (Wellman et al., 2001; Wellman and Liu, 2004; Kammermeier and Paulus, 2018; Priewasser et al., 2020). Indeed, we considered a correct answer to be when the child answered both questions (ToM and reality) correctly. Our participants were also randomly assigned to the “Robot” and “Human” conditions in a homogeneous way.

Replicating our procedure with children between 4 and 9 would be important and interesting in order to see if our methodology produces a similar improvement for the 4–6 years-old age group or if this level of performance corresponds to a plateau for children below the age of 6. In the first case, the traditional results found in the literature of explicit FBT, showing a progression with age, would not qualitatively change but simply be shifted toward younger age groups. It would then be essential to replicate our procedure with children between 2 and 3 to decide at what age the explicit FBT can start to become successful. In the second case, with a limited success rate before the age of 6, the 6–7 age group would be the pivotal age for reaching almost a 90% success rate with explicit FBT. This would imply that important pragmatic and/or cognitive capacities would still be lacking at the age of 5, preventing a total success at this age. This would not necessarily contradict our pragmatic approach. Indeed, numerous studies report that 6 is the pivotal age to be able to correctly generate relevance implicatures (Bosco and Gabbatore, 2017; Grigoroglou and Papafragou, 2017). As explained above, explicit FBT are complex as they require a “triple attribution of mental states” (Helming et al., 2014, 2016; Westra and Carruthers, 2017). They imply not only that the child must take into account the perspective of the character of the story but also that of their interlocutor, who is an adult experimenter in the standard test, since the child infers expectations from them and finally their own perspective. Consequently, this task would not be a first order task, but rather a second order task, thus explaining the threshold of a 60% success rate.

It is also interesting to look specifically at how the children responded to the control questions in our “mentor-child” context. As was shown by Perner and Wimmer (1985) the two types of success coding (with or without the reality question) slightly modify the results downwards without changing the interpretation: the chances of success in the “Robot” condition relative to that in the “Human” condition went from 4 times higher to about 3.5 times higher. The success rate decreased when the answer to the reality question was considered. In terms of proportions, both conditions had a similar success rate in the reality question (75% in “Human” and 77.7% in “Robot” conditions). This may confirm that the emphasis on the “slow” trait of the robot allows us to disambiguate a large part of the reality question. For the memory question, as predicted, we found a higher success rate (85%) in the “Robot” condition which is similar to the 83.7% observed in Wimmer and Perner (1983) with children between 4 and 5 years-old. This result seems to confirm our hypothesis that this question is noticeably ambiguous in the standard context for preschool children.

The fact that this “mentor-child” context works with 3 years-old children also provides new arguments in favor of the use of a humanoid robot as a tool in experimental research on children and adults. Our study did not have as its main objective to measure the importance of the robot tool itself but rather the influence of the context it allowed to produce. However, it would be important in a future study to see if there is a specific robot effect in our results that can stand out on its own. We can run the experiment with puppets or other objects representing an “ignorant,” “naive,” and “slow” entity (in an unpublished exploratory study on the class inclusion task Jamet, Saïbou-Dumont and Baratgin (2018) obtain, from children of French Guyana, similar performances to those obtained in Jamet et al. (2018) with the use of a puppet or a man disguised as a robot instead of the NAO robot^19^). A second possibility would be to run the study with a knowledgeable and intelligent “NAO teacher” in addition to the human experimenter and to the slow robot. Many studies have shown that children as young as 3 years-old accepted the NAO robot as a possible teacher (Rosanda and Istenic Starcic, 2020). Oranç and Küntay (2020) observed in children from 3 to 6 years-old a clear preference to ask the robot questions about machines, and less about biology and psychology. Thus, one could expect that in this situation children would be even less inclined to interpret the ToM question correctly as being a question about Maxi's beliefs. All this seems to indicate that our results are largely the consequence of the “mentor-child” context.

Our study also brings two important new elements on child-robot interaction. Firstly, our study seems to confirm that preschool children attribute beliefs to the robot as was also indicated in recent studies (Di Dio et al., 2018, 2020a; Marchetti et al., 2018). Secondly, in our study the child can behave like a mentor, with the motivation to help a robot understand a story. This helping behavior still happened even though physical interactions are quite limited. Indeed, the robot did not have a great autonomy of movement when seated in front of the child and it displayed few expressions (the NAO robot cannot smile and its facial expressions are very limited: only its eyes can change colors to signify an emotion). This is coherent with results from Martin et al. (2020a,b) which indicate that the helping behavior of children does not seem to be conditioned to the level of animated autonomy nor to the friendly expressions of the robot's voice.

While our methodology seems to work for an interaction with children older than 3 years and 2 months, children between 5 and 6 years-old, and also with adults (Masson et al., 2015, 2016; Masson et al., 2017a,b), children under the age of 3 did not agree to stay “alone” with NAO. It is possible that the choice of a humanoid robot may trouble young children. Di Dio et al. (2020b) shows that 3 years-old children tend to trust humans more than robots, as opposed to 7 years-old children. Manzi et al. (2020) showed that children of 5, 7, and 9 years-old differently assign mental states to two humanoid robots, NAO and Robovie, differing on their level of anthropomorphism. It is possible that, for very young children under 3 years-old, the NAO robot may not be the most adequate tool (see Damiano et al., 2015, for a review of the different types of robots). This would explain the low number of studies with children of this age. Recent reviews on the interactions between neuro-typical children and a robot (Jamet et al., 2018; Neumann, 2020; van Straten et al., 2020) indicate that only one study was conducted using NAO and a group of children from 2 to 8 years-old (Yasumatsu et al., 2017). The few other studies conducted on 2 years-old either used the tiny humanoid robot QRIO that is smaller than a 2 years-old child (Tanaka et al., 2007), the iRobiQ robot that looks more like a toy (Hsiao et al., 2015), or robots specifically designed to be enjoyed by young children like the stuffed dragon robot Dragonbot (Kory Westlund et al., 2017) and the RUBI-4 (Movellan et al., 2009). Thus, should we decide to do a longitudinal study from 2 to 9 years-old using our contextual procedure we would need to study which robot is the most relevant to play the role of a rather slow and ignorant being for all ages.

## 6. Conclusion

The essential proposition that has been developed and tested in our study is that the answer to the ToM question crucially depends on the “conversational logic” at play in the contextualized interactions between the experimenter and the child. This interaction shapes the child's interpretation of the question. Our contextual modification pragmatic filters the ToM question, removing irrelevant interpretations. The standard paradigm forces the child to perform a relevance search to interpret an ambiguous question asked by an expert (with a status like that of a teacher) within the limits of the child's own meta-cognitive knowledge. In our “mentor-child” context the child answers an unequivocal question about the beliefs of the protagonist of the story asked by a somewhat slow entity who needs their help. Here, the 3 years-old child can answer correctly even if their meta-cognitive knowledge is poorly developed. This procedure helps us become more “competent” speaker-experimenters (Sperber, 1994) as it offers a tool to place ourselves at the level of the young child's interpretation strategy. This allows them to realize what is relevant to answer the question correctly. For similar reasons we believe that this procedure may also help with the understanding of the second-order ToM (Perner and Wimmer, 1985). It could reduce the ambiguity of the question of the experimenter which exists in many experimental paradigms. Results of Lombardi et al. (2018) indeed indicate, using a dialogical perspective, that a considerable part of the supposed failures observed with children in the second order task are in fact the result of an adverse pragmatic context. In addition to the Piagetian tasks of length and number conservation (McGarrigle and Donaldson, 1974), volume conservation (Jamet et al., 2014), or class inclusion (Politzer, 2016), there are a variety of experimental paradigms that lend themselves well to our disambiguation methodology. The “mentor-child” context could also facilitate some studies with atypically developing participants, such as individuals with an Autism Spectrum Disorder who show both deficient performance on the false belief task (Baron-Cohen, 1997) and in language pragmatics (Angeleri et al., 2016). Finally, our methodology also offers new clues on the relevance of human-robot interaction, and in particular on child-robot interaction. More studies should most certainly focus on the interaction between children and robots, taking in consideration the beliefs they associate to these tools, and their effect on well-known psychological results.

## Data Availability Statement

The datasets analyzed for this study can be found in the Open Science Framework repository at the following address https://osf.io/ey4n5/?view_only=d8d2e16f39ea4186b994e2468a7408cd.

## Ethics Statement

The studies involving human participants were reviewed and approved by M. Charles El-nouty, Professeur des Universités en Mathématiques, LAGA UMR7539, Université Paris 13: President of the Committee. M. Jean-Yves Henry, Chirurgien-Dentiste diplômé de l'Université Paris 7; M. Michel Dionnet, Chef de cuisine, Membre titulaire de l'Académie Culinaire de France; M. Fabrice Gutnick, MCF associé en Sciences de l'Éducation, Université Jules Vernes Amiens, Psychologue du travail; Mme Dominique Klarsy Médecin du travail. Written informed consent to participate in this study was provided by the participants' legal guardian/next of kin.

## Author Contributions

JB and FJ: conceptual elaboration. JB, FJ, and MD-S: design of the study. MD-S and FJ: data collection. J-LS: data analysis. JB and MD-S: draft of the manuscript. JB, BJ, and FJ: critical revision of the manuscript. All authors contributed to the article and approved the submitted version.

## Conflict of Interest

The authors declare that the research was conducted in the absence of any commercial or financial relationships that could be construed as a potential conflict of interest.

## Footnotes

^1^The situation is more or less complex depending on the level of false belief evaluated. Three levels of representation (three gradual orders of difficulty) obtained at different ages can be distinguished (Duval et al., 2011). Order zero is automatically acquired. It corresponds to what we are currently thinking about. First order corresponds to the inference of the mental state of someone else and would only be acquired at the age of 4. The second order refers to the inference of the mental state of another person about another person and should be acquired between 6 and 7. This paper is mainly interested in the age at which children acquire the first order ToM. For the sake of simplicity, we will omit to specify “first order” when we refer to “explicit FBT” in the rest of this paper.

^2^In Wimmer and Perner (1983) Maxi would put the chocolate in the “blue” cupboard and his mother would move it to the “green” cupboard. We've interchanged the colors in this article to match the clips used in our experiment that were taken from Duval et al. (2011, p. 45).

^3^See for a recent argument in favor of this hypothesis (Doherty and Perner, 2020).

^4^Clues exist seeming to indicate that the late success in explicit FBT may indeed be the result of learning from repeated social experiences (Wang and Su, 2009). Studies show that a correlation exists between the number of brothers and sisters of a similar age and the comprehension of false beliefs (Perner et al., 1994; Ruffman et al., 2012; Jenkins and Astington, 2014). From the age of 3 the child can use language from a meta-cognitive point of view to lie (Lewis and Saarni, 1993) and start to be able to use contextual information (Salomo et al., 2013). It is necessary to wait the age of 4 to see children able to adapt their discourse by taking into account the age of the listener, their status and their gender; and can adapt their discourse to younger people. They can also ask a conversation partner to reformulate an utterance if they did not understand it (Clark and Amaral, 2010).

^5^These two additional interpretations were already evoked in (Perner et al., 1987, p. 126): “They may have misinterpreted the test question: ‘Where will the protagonist look for the chocolate?' as meaning, ‘Where should he look?' or ‘Help him to find it!”' These authors changed the question to “Where does he think the chocolate is?” However, as pointed out in Westra and Carruthers (2017), the term “think” requires more cognitive resources than the term “to look.” Also, this version complicates rather than simplifies the issue, which explains why it does not improve the performance of young children.

^6^Several pieces of experimental evidence indicate that 3 years-old children easily distinguish between what another person knows and does not (Hogrefe et al., 1986; Perner and Leekam, 1986).

^7^Young children seem to better understand their role in educational activities when they are explicitly formulated (Jeong and Frye, 2018a).

^8^It was only children 4 years-old (no younger children had answered the ToM question correctly).

^9^The reality question, in this context, looks like a violation of the principle of informativeness (Grice's Maxim of Quantity, 1975; Ducrot's law of exhaustiveness, 1980/2008) which requires that each participant in a conversation answer their partner's utterance with an appropriate quantity of information (neither too little nor too much). If multiple experimental clues question the complete acquisition of this principle at the age of 3 (Conti and Camras, 1984; Noveck, 2001; Eskritt et al., 2008), other studies indicate that some children of that age show skills like adapting their communicative behavior to the state of knowledge of their partners (O'Neill, 1996; Dunham et al., 2000; Ferrier et al., 2000). Perner and Leekam (1986) show that from the age of 3, children prefer mentioning first the most informative element and avoid mentioning elements already known by their listener.

^10^It can be noted that this interpretation of the memory question (an expectation of the experimenter to be controlling the answer to the ToM question) requires skills of second order ToM.

^11^Since the “ignorant,” “naive,” and “slow” robot was only there to strengthen the child's impression to be the one with the knowledge, its presence will be implied each time we refer to the “mentor-child” context.

^12^Written informed consent to participate in this study was provided by the participants' legal guardian/next of kin. All data was collected anonymously. The experiment was reviewed and approved by the Ethics Committee of the CHArt Laboratory. The Ethics statement can be obtained here: https://osf.io/wk4af?view_only=d8d2e16f39ea4186b994e2468a7408cd.

^13^The initial sample contained five classes of preschool children for a total of 70 children from 34 to 49 months-old. We had at least one 34 months-old child, one 35 months-old child, and so on, in each of the two conditions. During the experiment, we noticed that 3 children in the “robot” condition (the youngest: 34, 35, and 36 months-old) became really scared when the NAO robot started moving, lifting its head and looking at the child. The movement of the robot is not as fluid as that of a human, and the noise of the motors is quite noticeable. In consequence these three children had to be removed from the condition. In order to keep the conditions homogeneous in terms of age, we removed one child under 3 years-old in the “robot” condition who had succeeded in the task, and all 4 youngest children in the “human” condition, who had all failed in the task.

^14^In both conditions all children correctly named the two colors.

^15^A script of the interaction between a “mentor-child” and the NAO robot, written as a clinical and critical Piagetian interview (Ducret, 2016) can be found at https://osf.io/z5s7k/?view_only=d8d2e16f39ea4186b994e2468a7408cd.

^16^The companion insisted on this specific point.

^17^The complete R script of the analyses is available at https://osf.io/34hzn/?view_only=d8d2e16f39ea4186b994e2468a7408cd and the data itself is available at https://osf.io/wzx7g/?view_only=d8d2e16f39ea4186b994e2468a7408cd.

^18^Jamet et al. (2018) randomly assigned 40 children (between 5 and 6 years-old) to two conditions similar to those we created for the present study (“Human Experimenter” vs. “Ignorant NAO Robot”). The authors observed a clear improvement in performance in the “Ignorant NAO Robot” condition: one child out of five answered correctly in the “Human Experimenter” condition, and more than six children out of ten in the “Ignorant NAO Robot” condition.

^19^Experiments were carried out during the MIN formation of teachers requested by the rector of the academy of French Guyana.
